# Antiplasmodial Activity and Toxicological Assessment of Curcumin PLGA-Encapsulated Nanoparticles

**DOI:** 10.3389/fphar.2017.00622

**Published:** 2017-09-06

**Authors:** Zulaikha A. Busari, Kabiru A. Dauda, Olajumoke A. Morenikeji, Funmilayo Afolayan, Oyetunde T. Oyeyemi, Jairam Meena, Debasis Sahu, Amulya K. Panda

**Affiliations:** ^1^Department of Zoology, University of Ibadan Ibadan, Nigeria; ^2^Department of Biological Sciences, University of Medical Sciences Ondo, Nigeria; ^3^Product Development Cell, National Institute of Immunology New Delhi, India; ^4^Department of Biochemistry, School of Bioengineering and Biosciences, Lovely Professional University Phagwara, India

**Keywords:** polymeric nanoparticles, curcumin, antimalarial, safety

## Abstract

Curcumin is a polyphenolic pigment isolated from the rhizomes of *Curcuma longa* (turmeric), a medicinal plant widely used in the ancient Indian and Chinese medicine. The antiplasmodial activity of curcumin is often hampered by its fast metabolism and poor water solubility, thus its incorporation into a delivery system could circumvent this problem. This study aimed to evaluate the *in vivo* antiplasmodial activity and the toxicity assessment of curcumin incorporated into poly (lactic-co-glycolic) acid (PLGA) nanoparticles. Curcumin was loaded with poly (D,L-lactic-co-glycolic acid) (PLGA) using solvent evaporation from oil-in-water single emulsion method. The nanoparticles were characterized and evaluated *in vivo* for antimalarial activities using Peter’s 4-day suppressive protocol in mice model. Hematological and hepatic toxicity assays were performed on whole blood and plasma, respectively. *In vivo* anti-parasitic test and toxicity assays for free and encapsulated drug were performed at 5 and 10 mg/kg. *In vitro* cytotoxicity of free and PLGA encapsulated curcumin (Cur-PLGA) to RAW 264.7 cell line was also determined at varying concentrations (1000–7.8 μg/mL). The size and entrapment efficiency of the nanoparticulate drug formulated was 291.2 ± 82.1 nm and 21.8 ± 0.4 respectively. The percentage parasite suppression (56.8%) at 5 mg/kg was significantly higher than in free drug (40.5%) of similar concentration (*p* < 0.05) but not at 10 mg/kg (49.5%) at 4-day post-treatment. There were no significant differences in most of the recorded blood parameters in free curcumin and PLGA encapsulated nanoparticulate form (*p* > 0.05) except in lymphocytes which were significantly higher in Cur-PLGA compared to the free drug (*p* < 0.05). There were no significant differences in hepatotoxic biomarkers; aspartate aminotransferase and alanine aminotransferase concentrations in various treatment groups (*p* > 0.05). At higher concentrations (1000 and 500 μg/mL), Cur-PLGA entrapped nanoparticle showed higher toxicity compared with the free drug (*p* < 0.05) in exposed RAW 264.7 cell line. The cell viability was, however, higher in Cur-PLGA nanoparticles than in free curcumin at lower concentrations (*p* > 0.05). The antiplasmodial activity and safety of Cur-PLGA was better at lower concentration.

## Introduction

Malaria poses a major public health threat in the developing world with an estimated 243 million new cases reported toward the end of the last decade. An estimated 863,000 deaths are recorded worldwide (World Health Organization [WHO], 2009). The most pathogenic species of the five human malaria parasites is *Plasmodium falciparum*, followed by *Plasmodium vivax* which causes most morbidity and latent infection (Anstey et al., 2009). Early detection of infection and treatment is cardinal to abating malaria problem in sub-Saharan Africa, however, effective curtailment of malaria becomes very difficult owning to growing resistance of *P. falciparum* to drugs once effective (Isacchi et al., 2012).

Research efforts toward the discovery of anti-parasitic agents from plants are on the increase. One of these plants is *Curcuma longa* from which curcumin [1,7-bis(4-hydroxy-3-methoxyphenyl)-1,6-heptadiene-3,5-dione] is derived. Curcumin has been widely exploited for its anti-inflammatory, anti-infectious, and anticancer activities (Ruby et al., 1995; Han et al., 2002; Maheshwari et al., 2006). It has also been reported to be efficacious against parasitic protozoans such as *Giardia*, *Leishmania*, and trypanosomes, and parasitic helminth such as *Schistosoma mansoni* (Nose et al., 1998; Pérez-Arriaga et al., 2006; Allam, 2009; Magalhães et al., 2009; Silva et al., 2009). In a similar vein, the nanoparticulate form of curcumin has been tested on different diseases of which showed favorable results, e.g., anticancer (Duan et al., 2010; Anuchapreeda et al., 2012) and anti-*Schistosoma* (Luz et al., 2012).

Despite the numerous application of curcumin, its clinical development has been hampered due to its fast metabolism and poor water solubility (Tonnesen, 2002). Its lipophilic nature results in low bioavailability, degradation at high pH, and photodegradation (Duan et al., 2010). To increase the solubility and bioavailability of curcumin for improved efficacy, it has always been incorporated into different delivery systems. Curcumin has been encapsulated in polymeric nanoparticles, lipid-based nanoparticles, liposome, chitosan/poly (butyl cyanoacrylate) nanoparticles, and biodegradable microsphere (Bisht et al., 2007; Tiyaboonchai et al., 2007; Sou et al., 2009; Duan et al., 2010).

While delivery system like liposome has been shown to be toxic (Chen et al., 2009), polymeric nanoparticles have the advantage of low toxicity and therefore have been approved by the Food and Drug Administration (FDA). Poly (lactic-co-glycolic) acid (PLGA) nanoparticles have been used as effective drug delivery systems for the controlled release of various pharmacologically active moieties such as artesunate (Nguyen et al., 2015) and curcumin (Yallapu et al., 2010; Luz et al., 2012). Although curcumin-encapsulated nanoparticles have gained wide application in the treatment of diverse diseases and its free form used against malaria, very few studies have observed the antiplasmodial potency of the nanoparticulate form of curcumin (Nayaka et al., 2010). More so, due to some evidences on relative toxicity of curcumin (Kunwar et al., 2009; Palanikumar et al., 2011), a comparison on toxicological evaluation of the main compound and its nanoparticles derivative is necessary. The aim of this study was therefore to evaluate the *in vivo* antiplasmodial activity and toxicological assessment of curcumin incorporated into PLGA nanoparticles.

## Materials and Methods

### Materials

Curcumin (from *C. longa* Linn), polyvinyl alcohol (PVA) (M_W_ = 30–70 kDa), D-mannitol, dimethyl sulfoxide (DMSO), and 3-(4,5-dimethylthiazolyl-2)-2,5-diphenyltetrazolium bromide (MTT) were purchased from Sigma–Aldrich (St. Louis, MO, United States). Dichloromethane and acetone were procured from Merck Serono Ltd. Poly (D,L-lactic-*co*-glycolic acid) (PLGA) (intrinsic viscosity η = 0.41 dL/g, copolymer ratio 50:50, 45 kDa) was purchased from Purac Biochem, Holland. Water purified by Milli-Q_plus_ system from Millipore (MQ water) was used. Culture medium RPMI-1640, fetal calf serum (FCS), and antibiotic–antimycotic were obtained from GIBCO Invitrogen (Grand Island, NY, United States). All other chemicals were of analytical grade.

### Formulation of Nanoparticles

The formulation of curcumin encapsulated with poly (D,L-lactic-*co*-glycolic acid) (PLGA) nanoparticle was done using solvent evaporation from oil-in-water single emulsion according to the method described by Chittasupho et al. (2009). Briefly, curcumin (5 mg) and PLGA (50 mg) constituting 1:10 drug/polymer ratio were dissolved in organic solvents comprising 3.5 mL of dichloromethane and 0.5 mL of acetone. The organic phase was then added drop-wise to 16 mL aqueous solution (2% PVA as emulsifier) with sonication at 30 W, 40% duty cycle for 3 min in ice cold water. The emulsion was repeatedly stirred until all organic solvents evaporated. The formulation was centrifuged at 16,000 × *g* for 15 min and then washed three times. Dry powders were obtained by lyophilization of frozen samples in the presence of 5% mannitol as cryoprotectants.

### Particle Size Measurement and Zeta Potential

Homogenous solution of dried powder of Cur-PLGA nanoparticles was made in a MQ water and was then transferred to a transparent sizing cuvette for particle size and polydispersity index (PDI) measurement using a Zetasizer Nano-ZS (Malvern Instruments, United Kingdom). Zeta potential was measured using a clear zeta cell. All measurements were taken in triplicate and results were expressed as mean ± SD.

### Scanning Electron Microscopy (SEM)

Sample preparation for nanoparticle surface morphology as examined by scanning electron microscope (SEM) was carried out by the method described by Khuroo et al. (2014). Briefly, a small quantity of formulated nanoparticles was suspended in MQ water and then mounted on metallic stubs using double-sided carbon adhesive tape. A circular cover-slip was gently placed over the stub to enable even distribution of the sample suspension. To facilitate uniform conductivity, a silver paint lining was applied to the edges of the cover-slip to fill the narrow spacing between the stub and the cover-slip. They were viewed with an EVO LS 10 (Carl Zeiss, Brighton, Germany) SEM which operates at an accelerating voltage of 20 kV under high vacuum. The particles were examined for surface characteristics like shape, and presence of aggregation.

### X-Ray Diffraction (XRD) Analysis and Differential Scanning Calorimetry (DSC)

X’Pert-PRO multipurpose X-ray diffractometer (PANalytical, Netherland) was used to generate the X-ray diffraction (XRD) patterns of the lyophilized Cur-PLGA entrapped nanoparticles. The CuKα radiation was generated at 45 kV and 40 mA in the diffraction angle range of 5–40° 2𝜃.

The physical state of the formulated nanoparticles was obtained by differential scanning calorimetry (DSC) studies. Thermogram was obtained using DSC PerkinElmer Pyris 1 (United States). The DSC cell was purged using dry nitrogen gas at a flow rate of 40 mL/min. Lyophilized Cur-PLGA entrapped nanoparticles (6–8 mg) were sealed in a standard aluminum pan with lid and heated at a rate of 5°C/min from 50 to 300°C.

### Drug Entrapment and Encapsulation Efficiency

Drug entrapment and encapsulation efficiency were assessed by a modified method of Anitha et al. (2011a). Ten milligrams (10 mg) of lyophilized nanoparticles was dissolved in 1 mL acetonitrile. The solvent from the properly dissolved homogenous solution was evaporated for 9–10 h at 50°C using heater (CH-100, Biosan Ltd.). Residue was suspended in 500 μL methanol, followed by vortexing and centrifuging at 13,000 × *g* for 20 min. Supernatant fluid (500 μL) collected after centrifugation was stored. The process was repeated with 500 μL of acetonitrile.

A stock solution (100 μg/mL) of curcumin was prepared from dissolution of 5 mg of the drug in 5 mL of absolute methanol. Free curcumin concentrations were determined using UV–vis spectrophotometer (Ultrospec^®^ 2100 *pro*, Amershan Biosciences) at λ_max_ 446 nm. A standard curve from UV–vis spectrophotometer analyses was obtained from graded curcumin concentrations ranging from 5 to 70 μg/mL. The stored supernatant containing encapsulated curcumin was analyzed by the same method. Then the drug content and encapsulation efficiency of the formulation were estimated.

### *In Vitro* Release Kinetics of Drug-Entrapped Nanoparticles

Ten milligrams (10 mg) of Cur-PLGA entrapped nanoparticles was suspended in 10 mL PBS, pH 7.4 (Anitha et al., 2011b). The mixture was incubated in a rotary shaker at 200 × *g*. The sample was centrifuged at 16,000 × *g* for 10 min at specific time interval after which 1 mL of supernatant was withdrawn and then replaced with 1 mL of fresh PBS.

Five milligrams (5 mg) of lyophilized free curcumin was dissolved in 5 mL absolute methanol to form a stock solution (100 μg/mL). The working standard concentrations ranging from 5 to 70 μg/mL were prepared from the stock using PBS (pH 7.4) as the diluent. The UV-absorbance was measured at wavelength 446 nm. The UV-absorbance analysis of the supernatant from Cur-PLGA entrapped nanoparticles was also carried out at different time interval. The *in vitro* curcumin release from Cur-PLGA was estimated from the standard plot obtained from UV-absorbance analysis of free curcumin.

### Mice Model and Parasite Strain

All drug testing involving the use of experimental animals adhered to the Principles of Laboratory Animal Care (NIH publication #85-23, revised in 1985). The protocol was approved by the Animal Care Use and Research Ethics Committee (ACUREC) of the University of Ibadan, Nigeria (UI-ACUREC/17/0067). Swiss male albino mice were obtained from the Animal House Center of the Department of Pharmacology, University of Ibadan, Nigeria. *Plasmodium berghei* NK-65 was obtained from Institute of Advanced Medical Research and Training (IAMRAT), University College Hospital, University of Ibadan, Nigeria.

### Antiplasmodial Evaluation with Peters’ 4-Day Suppressive Test

A total of 25 Swiss male albino mice (20.0 ± 2.0 g) 5–6 weeks old were used in a Peters’ 4-day suppressive test giving five groups of five mice. Animals were maintained in standard pathogen-free conditions and fed *ad libitum*. *P. berghei* infected red blood cells obtained from an infected donor Swiss male mice was diluted in physiological saline to 1 × 10^7^ pEry/mL. Intraperitoneal (ip) mice infection was done with an aliquot of 0.2 mL of the parasites suspension. The Cur-PLGA entrapped nanoparticles and free curcumin drug were reconstituted in 10% Tween 80 at different concentrations (5 and 10 mg/kg). The mice were treated orally with 0.2 mL of the drugs’ suspensions 2-h post-infection. The negative control group received 0.2 mL of the vehicle while the positive control group received oral dose of 4 mg/kg free artesunate (Chinaeke et al., 2015). Animals were further treated for 3 days. At the 4th day, after infection, blood smears were made from the tail vein, Giemsa-stained, and examined microscopically in immersion oil (Cheesbrough, 1998). Infected erythrocytes were counted per total erythrocytes in four fields and the percentage *P. berghei* suppression was calculated at the 4th day after treatment.

### Changes in Weight and Rectal Temperature

The body weight and rectal temperature of mice with mean *P. berghei* density before treatments were determined. The same were repeated 4-day post-treatment with free curcumin and Cur-PLGA encapsulated nanoparticles.

### *In Vivo* Toxicity Assays

Acclimatized albino rats weighing 94–105 g were administered 5 and 10 mg/kg free and encapsulated curcumin for 4 days. Two milliliters (2 mL) of blood samples obtained through retro-orbital puncture was collected into EDTA tubes. Packed cell volume (PCV), red blood cells and white blood cell counts, hemoglobin level, eosinophils, and neutrophils were determined by standard procedures. Liver enzymes, alanine aminotransferase (ALT) and aspartate aminotransferase (AST), were determined by Randox Diagnostic Kits.

### Cell Culture and *In Vitro* Cytotoxicity Assay

Murine RAW 264.7 macrophages obtained from Product Development Cell-1 Laboratory, National Institute of Immunology, New Delhi were used for the *in vitro* cytotoxicity assay. The cells were incubated in RPMI 1640 (Sigma) containing 10% FCS and 1% PSA at 37°C and 5% CO_2_ in an air humidified incubator. Cells were harvested when reached 80–90% confluence and were washed with RPMI medium. After dilution of cells to 5 × 10^4^ cells/mL, 200 μL of the cell suspension was seeded per well in sterile 96-well plates and incubated for 24 h to allow cell attachment.

The seeded RAW 264.7 cells were incubated with free drug and drug-loaded PLGA nanoparticles suspension at concentrations ranging from 7.8 to 1000 μg/mL for 24 h (Zhanga and Feng, 2006). The medium was removed and then replaced with 100 μL of culture medium and 10 μL of MTT (5 mg/mL in PBS) at designated time intervals. After 3–4 h incubation, the culture solution was removed, leaving behind Formazan crystals precipitate. One hundred microliters (100 μL) of DMSO was added to each well and the plate was then read by the microplate reader at 570 nm. Cell viability was calculated and the IC_50_ was determined.

### Statistical Analysis

Data were analyzed using GraphPad Prism 6 (GraphPad Software, Inc., La Jolla, CA, United States). Two-way analyses of variance (ANOVA) and Tukey’s multiple comparison tests were used to test for significant differences. Statistical significance was determined at *p* < 0.05. Cytotoxicity was expressed as mean inhibition relative to the unexposed control ± standard deviation (SD) for three parallel readings.

## Results

### Formulation, Characterization, and Release Kinetics of Nanoparticle

**FIGURE 1:**
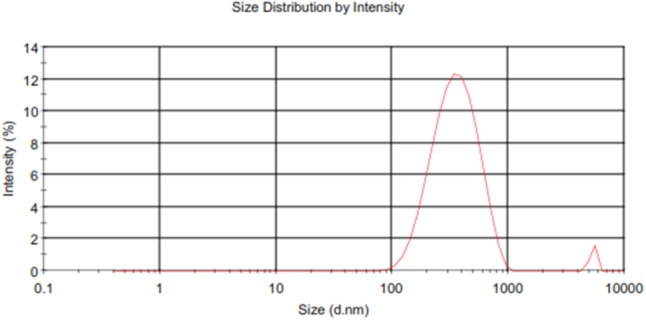
Size distribution of Cur-PLGA nanoparticles.

**FIGURE 2:**
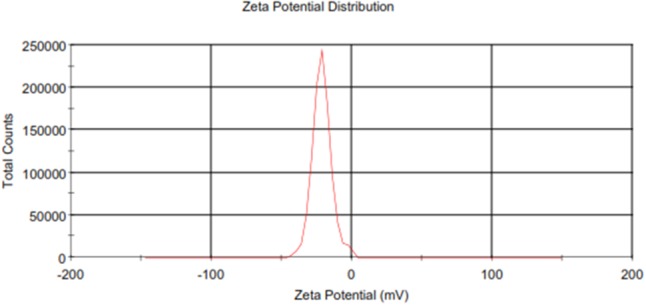
Zeta potential distribution of Cur-PLGA nanoparticles.

**FIGURE 3:**
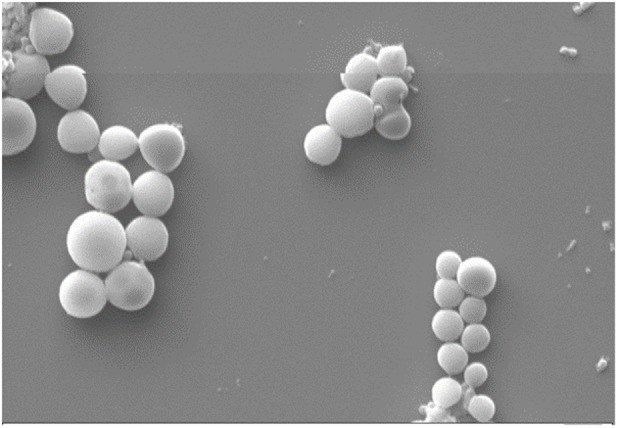
Scanning electron microscopy of Cur-PLGA.

The particle size, zeta potential, PDI, and entrapment efficiency (%EE) of Cur-PLGA entrapped nanoparticles were 291.2 ± 82.1 nm, -14.4 ± 5.8 mV, 0.146 ± 0.09, and 21.8 ± 0.4, respectively. The particle size and zeta potential distribution patterns were presented in **Figures 1, 2**. The SEM image analyses of the particles showed smooth surfaces with spherical morphology (**Figure 3**).

**FIGURE 4:**
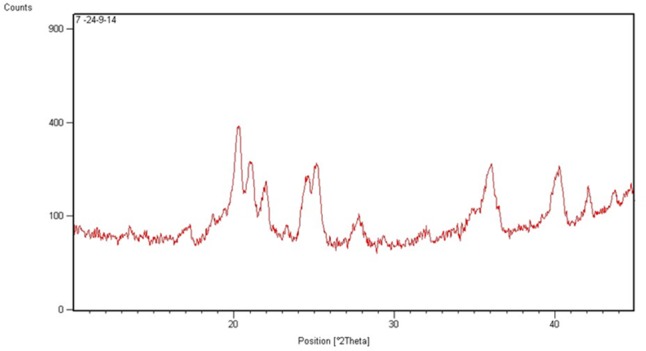
XRD spectra of Cur-PLGA nanoparticles.

**FIGURE 5:**
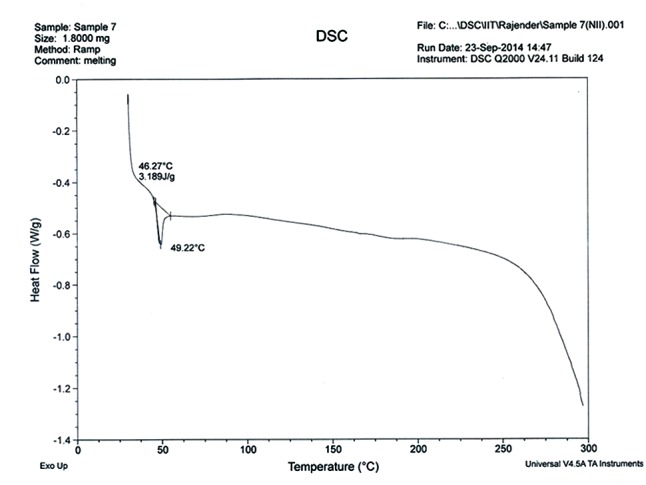
Thermograms of Cur-PLGA entrapped nanoparticles.

**FIGURE 6:**
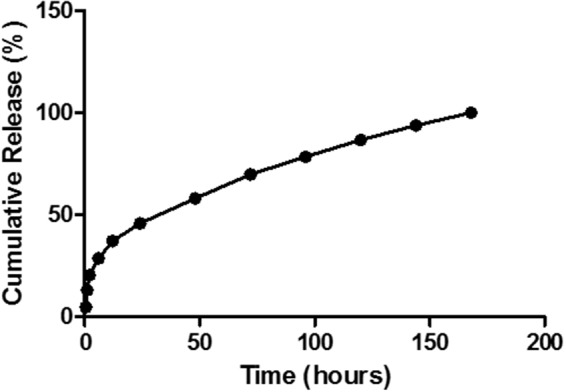
Curcumin *in vitro* release from PLGA.

The P-XRD diffractograms of Cur-PLGA showed characteristic intensity peaks visible at 14.7, 18.9, 20.6, 21.3, 22.3, 23.6, 24.8, 25.4, 28.1, 26.1, 40.5, and 42.1° (**Figure 4**). The DSC thermogram of PLGA entrapped curcumin was shown in **Figure 5**. The endothermic melting peak of Cur-PLGA nanoparticle was 49.2°C. An initial burst corresponding to about 13.5% of the total drug release was observed within 1 h followed by a sustained released in Cur-PLGA entrapped nanoparticles (**Figure 6**).

### Parasite Suppression, Weight, and Rectal Temperature Variations

There was no difference in parasite suppression (49.5%) at 10 mg/kg of free curcumin and equivalent concentration of its nanoparticulate form (*p* > 0.05). However, at a lower concentration 5 mg/kg, percentage parasite suppression (56.8%) was significantly higher than in free drug (40.5%) (*p* < 0.05) at 4-day post-treatment. The negative control group showed no parasite reduction (**Table 1**). The body weight of mice decreased after treatment although the weight reduction was not significant (*p* > 0.05). The same was observed for rectal temperature (**Table 1**).

**Table 1:** Response to treatment in Swiss Albino mice.

Dose	Parasitemia ±*SD*	Parasites suppression (%)	Weight (g)		Temperature (°C)	
			Before	After	Before	After
Cur-free (5 mg/kg)	2.7 ± 1.6	40.5^a^	22.0 ± 3.3	17.5 ± 4.2	34.8 ± 2.0	36.0 ± 2.4
Cur-free (10 mg/kg)	2.3 ± 0.6	49.5^a^	20.6 ± 1.6	18.2 ± 3.7	34.8 ± 0.5	35.0 ± 0.8
Cur-PLGA (5 mg/kg)	2.0 ± 0.1	56.8^b^	20.8 ± 2.3	19.0 ± 1.7	33.4 ± 0.6	34.3 ± 1.0
Cur-PLGA (10 mg/kg)	2.3 ± 0.2	49.5^a^	20.6 ± 1.6	18.2 ± 4.0	34.8 ± 0.5	35.0 ± 0.9
Artesunate (4 mg/kg)	1.9 ± 0.7	58.2^b^	28.0 ± 3.2	26.0 ± 1.3	34.5 ± 1.0	36.8 ± 1.0
Negative control	4.5 ± 0.6		24.2 ± 3.2	23.2 ± 1.2	36.1 ± 0.5	36.3 ± 1.0


*Similar superscripts denote no significant difference while different superscripts mean there is significant difference*.

### Hematological and Hepatic Toxicity Studies

**FIGURE 7:**
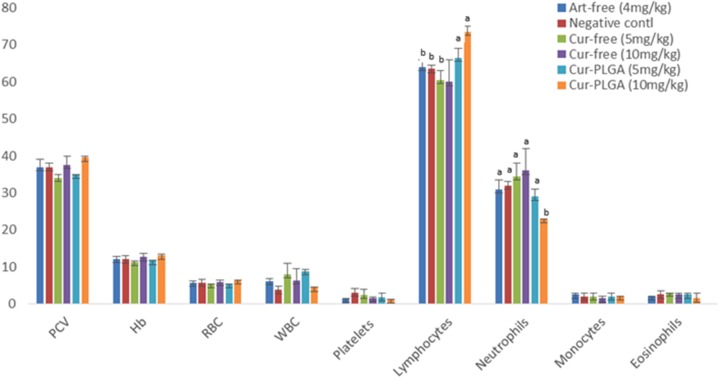
Hematological variations between free and nano-synthesized curcumin. WBC is expressed as ×10^5^ of the given values.

**FIGURE 8:**
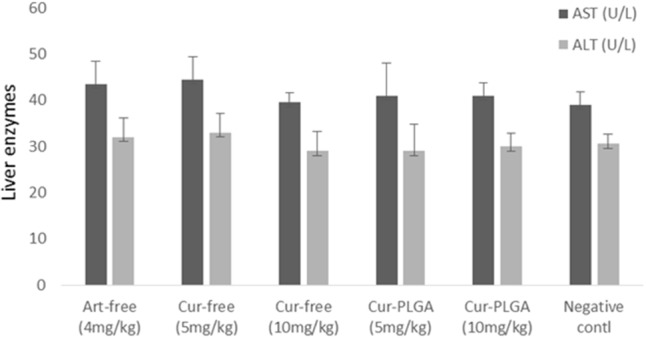
Hepatic toxicity assessment of free and nanoparticulate curcumin.

There were no significant differences in most of the recorded blood parameters in free curcumin and PLGA encapsulated nanoparticulate form (*p* > 0.05). The neutrophil counts were significantly higher in free drug of varying concentrations and in 5 mg/kg of Cur-PLGA nanoparticles than in 10 mg/kg Cur-PLGA nanoparticles. Lymphocyte counts in 5 (66.5 ± 2.5) and 10 mg/kg (73.5 ± 1.5) in Cur-PLGA nanoparticles were significantly higher than in corresponding 5 (60.5 ± 2.5) and 10 mg/kg (60.0 ± 6.0) of free curcumin (**Figure 7**) (*p* < 0.05). There were no significant differences in hepatotoxic biomarkers AST and ALT concentrations in various treatment groups (*p* > 0.05) (**Figure 8**).

### *In Vitro* Cytotoxicity Assay

**FIGURE 9:**
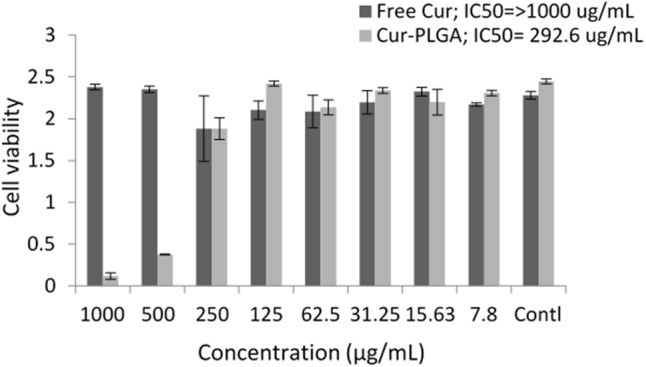
Cell viability and inhibitory concentrations in free and PLGA nanoparticle entrapped drug formulations.

At higher concentrations (1000 and 500 μg/mL), Cur-PLGA entrapped nanoparticles showed higher toxicity compared to the free drug (*p* < 0.05). The cell viability was, however, higher in Cur-PLGA nanoparticles than in free curcumin at lower concentrations (*p* > 0.05) (**Figure 9**). The IC_50_ of Cur-PLGA (292.6 μg/mL) was lower than that of free curcumin (1000 μg/mL).

## Discussion

A simple method of solvent evaporation was used to incorporate curcumin into PLGA nanoparticles to produce drug which readily dissolves in water. The Cur-PLGA nanoparticles gave a clear, well dispersed formulation in aqueous solution while native curcumin is poorly soluble in water with undissolved flakes of the compound visible in the solution (Mohanty and Sahoo, 2010). This, and its instability in physiological pH is a major challenge hampering its therapeutic applications (Ma et al., 2008; Shaikh et al., 2009).

At the early stage of the process, the polymer chain comprising the organic phase and the solvent diffuse into the aqueous phase (dispersing medium) (Luz et al., 2012). Desolvation of polymer resulting from the interaction between the solvent and the water leads to polymer precipitation, curcumin entrapment, and eventual formation of nanoparticles (Bilati et al., 2005).

Curcumin-PLGA nanoparticulate sizes observed in our study were similar to 200–220 nm curcumin loaded dextran sulfate–chitosan nanoparticles sizes range reported by Anitha et al. (2011a), our observed entrapment efficiency (21.8 ± 0.4) was lower than the later who reported approximately 74%. The low PDI recorded for the Cur-PLGA nanoparticles denotes its monodispersibility nature (Luz et al., 2012). The 13.5% burst release of the nanoparticles within 1 h suggests its therapeutic advantage as a sufficient amount of curcumin is rapidly released from the PLGA into the blood stream to exert an initial therapeutic effect followed by a controlled release of the remaining curcumin from the PLGA (Tiyaboonchai et al., 2007).

The diffractogram patterns showed that the entrapped drug was present in crystalline form. This crystalline nature has been suggested to be due to entrapment of drug crystals in the nanoparticles; the effect which is more pronounced in larger particle sizes (Panyam et al., 2004). The absence of decomposition exotherm in Cur-PLGA nanoparticles is an indication of physical stability while the occurrence of a thermogram peak showed some levels of chemical interaction in the drug formulation (Chadha et al., 2012; Chinaeke et al., 2015).

With increase reports on emergence of drug resistance to malaria’s mainstay drugs, new alternatives for the management of malaria are necessary. *In vitro* and *in vivo* studies on curcumin have proved its potential as antimalarial (Nandakumar et al., 2006; Martinelli et al., 2008). Nevertheless, its low bioavailability and rapid metabolism impair its full ethno-botanical benefits (Anand et al., 2007). Delivery systems are known to enhance its medicinal values. The study showed an improvement in antiplasmodial activity of the formulation at 5 mg/kg when compared to free curcumin. Prolonged circulation of curcumin encapsulated nanoparticles and improved bioavailability and absorption influenced by the small particle size could have been responsible for this observation. This is similar to a report on curcumin-loaded hydrogel nanoparticles (Dandekar et al., 2010). Better efficacy at lower concentration of curcumin nanoparticles was also reported in the same study. The increase in efficacy with increase in concentration of free drug is similar to an earlier report (Neto et al., 2013) but poses a disadvantage as this could result in drug-induced stress (Dandekar et al., 2010). The observed weight loss which was more pronounced in free drug groups and Cur-PLGA nanoparticles at 10 mg/kg may not be unconnected to drug-induced stress resulting in low daily food consumption coupled with increase parasite burden in the groups.

The lack of significant difference in most of the observed hematological parameters in free and encapsulated curcumin supports the claim that curcumin nanoparticles are non-toxic to blood cells. It is important, however, to note that the formulation at 5 and 10 mg/kg significantly improved hematopoietic properties due to increased percentage lymphocytes. This hematological observation also corroborated well with lack of significant difference in the expression of biochemical markers of hepatic toxicity; an observation which has been previously reported (Dandekar et al., 2010; Jana et al., 2014).

The observation that there was a higher toxicity of Cur-PLGA nanoparticle at very high concentrations (500 and 1000 μg/mL) when compared with free curcumin was noteworthy. Many studies on *in vitro* toxicity of curcumin nanoparticles have been targeted on cancer cells with very scarce information on normal cell lines. This bias might probably be due to the unverified assumption that curcumin nanoparticles are nontoxic due to the non-toxicity results often obtained with the free drug. Our observation was supported by the higher toxicity of liposomal curcuminoid (lipid entrapped curcumin) on human lymphocytes and splenocytes compared with free curcumin (Chen et al., 2009). It is unlikely that the toxicity observed in the study was caused by liposome as liposomal induced toxicity was knocked out by appropriate integration of liposomal curcumin with L-α-dimyristoylphosphatidylcholine (DMPC), L-α-dimyristoylphosphatidylglycerol (DMPG), and cholesterol (DMPC:DMPG:cholesterol: curcumin = 7:1:8:0.5 molar ratio). The higher toxicity of Cur-PLGA nanoparticles in *in vitro* may be due to increase in stability and decrease in aggregation (Sampedro et al., 1994) of the particles thus increasing its cellular uptake and increase toxicity in the cells especially at very high dosage.

Curcumin-loaded polymeric-based nanoparticle was successfully synthesized. The method of formulation is simple and reproducible. The formulated nanoparticulate drug showed higher potency against malaria parasite when compared with the free non-entrapped curcumin. More so, antimalarial activity of the drug is better at lower concentration; giving an advantage of reducing high dose drug-associated stress. The *in vivo* toxicity studies confirmed the safety of the formulated drug at tested dosages. It is important to administer the formulated drug at lower concentration due to significantly higher toxicity observed in the *in vitro* assay at very high concentrations. The long-term administration of low dosage of the formulated curcumin nanoparticles can prevent parasite recrudescence due to reported safety in the *in vivo* assays.

## Author Contributions

OO, FA, JM, AP, and OM conceived and designed experiments; KD, ZB, FA, OO, JM, and DS performed the experiments; AP contributed reagents/materials/analysis tools. OO analyzed the data and wrote paper first draft; and AP, JM, DS, FA, and OM revised the manuscript.

## Conflict of Interest Statement

The authors declare that the research was conducted in the absence of any commercial or financial relationships that could be construed as a potential conflict of interest.
